# Location‐Specific Hematoma Volume Predicts Early Neurological Deterioration in Supratentorial ICH

**DOI:** 10.1002/acn3.70351

**Published:** 2026-02-22

**Authors:** Zuoqiao Li, Guilin Meng, Zijie Wang, Xiao Hu, Jing Cheng, Chu Chen, Tiannan Yang, Jiaxin Luo, Zizhen Meng, Xueyun Liu, Qi Li

**Affiliations:** ^1^ Department of Neurology The First Affiliated Hospital of Chongqing Medical University Chongqing China; ^2^ Department of Neurology, Tongren Hospital Shanghai Jiao Tong University, School of Medicine Shanghai China; ^3^ Department of Neurology The Second Affiliated Hospital of Anhui Medical University Hefei China; ^4^ Department of Neurology and Neurosurgery The Third Affiliated Hospital of Chongqing Medical University Chongqing China

**Keywords:** hematoma volume, intracerebral hemorrhage, location, neurological deterioration, outcome

## Abstract

**Objective:**

Early neurological deterioration (END) adversely affects outcomes in patients with intracerebral hemorrhage (ICH). This study aimed to determine the location‐specific hematoma volumes for END in supratentorial ICH patients.

**Methods:**

We retrospectively analyzed supratentorial ICH patients presenting from two prospective cohorts. END was defined as a ≥ 4‐point increase in the National Institutes of Health Stroke Scale (NIHSS) score or a ≥ 2‐point decrease in the Glasgow Coma Scale score within 24 h of admission. The training cohort was used to determine location‐specific hematoma volume cutoffs for END and to develop the location‐specific hematoma volume for early neurological deterioration (LIVED) score. Internal validation used 30% of the cohort, with external validation in a separate cohort.

**Results:**

A total of 1199 patients with supratentorial ICH were included, divided into training (*n* = 633), internal validation (*n* = 272), and external validation (*n* = 294) cohorts. Hematoma volume thresholds for END were 21 mL for basal ganglia, 12 mL for thalamus, and 32 mL for lobar hemorrhages. Multivariable logistic regression identified location‐specific hematoma volume, right‐sided ICH, prior ischemic stroke, and NIHSS score as independent predictors, forming the LIVED score (range 0–5 points). The LIVED score showed superior predictive performance for END compared with established ICH scores, with the highest area under the curve (AUC) across cohorts. Additionally, it exhibited strong discrimination for 3‐month outcomes, including functional independence, poor outcomes, and mortality, with AUCs > 0.70 in all cohorts.

**Interpretation:**

Location‐specific hematoma volume thresholds independently predicted END, and the LIVED score demonstrated reliable performance for risk stratification in supratentorial ICH.

## 1 Introduction

Spontaneous intracerebral hemorrhage (ICH) accounts for about 30% of all strokes but disproportionate higher morbidity and mortality rates [1]. More than 30% of patients die within the first month and many survivors experiencing profound long‐term neurological disabilities [2, 3]. Early neurological deterioration (END) is not uncommon among ICH patients and occurs in approximately one‐quarter of ICH patients [4]. It is associated with elevated mortality and long‐term disability, resulting in a substantial burden on the healthcare system [5, 6]. Thus, identification of END tools are urgently needed to guide patient management and optimize individual therapy.

Hematoma volume has consistently emerged as a crucial determinant of clinical outcomes [7]. Larger hematomas typically exert greater mass effect, elevate intracranial pressure, and lead to more extensive disruption of neighboring brain regions, resulting in poorer functional outcomes [1, 8]. Recent studies suggested that the relationship between hematoma size and prognosis varied by location, and location‐specific volume thresholds might better predict clinical outcomes [9, 10, 11, 12]. For instance, hemorrhages in critical regions like the basal ganglia or thalamus, even when small, can cause severe neurological deficits, whereas larger hematomas in less critical areas may be more tolerable. While the concept of location‐specific hematoma volume tolerance is well recognized, prior studies have largely focused on functional outcomes or mortality. Data specifically addressing END in this context remain limited.

END is a critical event that occurs in the early stage of ICH and significantly impacts patient prognosis [13]. Early identification of patients at risk for END is essential for timely therapeutic interventions aimed at preventing further neurological decline. Existing ICH scoring systems primarily focus on outcomes at 3 month or beyond, lacking predictive accuracy for early events such as END [14, 15]. Therefore, we aimed to determine location‐specific hematoma volume thresholds and to derive an admission‐based risk score for the early prediction of neurological deterioration in patients with supratentorial ICH.

## 2 Methods

### 2.1 Study Design

This study was a retrospective analysis of prospectively collected data of supratentorial ICH patients admitted to the First Affiliated Hospital of Chongqing Medical University (CQMU1H cohort) from January 2016 to December 2022, and those admitted to the Second Affiliated Hospital of Anhui Medical University (AHMU2H cohort) from August 2022 to May 2024. The two cohorts were approved by the institutional review boards, respectively. Written informed consent was waived due to the retrospective design of the study. All study procedures were conducted in accordance with the Declaration of Helsinki and applicable local regulations. We randomly divided the CQMU1H cohort dataset into a training set (70%) and an internal validation set (30%), and utilized the AHMU2H cohort database for an independent external validation set to evaluate the scoring system.

### 2.2 Subjects

Subjects were consecutive patients admitted to the two centers with a diagnosis of primary ICH. Eligible participants met the following inclusion criteria: (1) age older than 18 years, and (2) supratentorial ICH confirmed by computed tomography (CT) imaging. The exclusion criteria were as follows: (1) symptom onset to admission CT of > 24 h; (2) presence of primary intraventricular hemorrhage (IVH) or ICH resulting from trauma, brain tumor, hemorrhagic transformation of a cerebral infarction, vascular abnormalities, or any other suspected secondary cause; (3) a baseline modified Rankin Scale (mRS) score of 4–5 prior to the occurrence of ICH; and (4) unavailability of data on END or 90‐day follow‐up functional outcomes.

### 2.3 Outcomes

The primary outcome of interest was END, defined as an increase of ≥ 4 points in the National Institutes of Health Stroke Scale (NIHSS) score or a decrease of ≥ 2 points in the Glasgow coma scale (GCS) score within the first 24 h of admission [13]. Functional outcomes at 90 days were assessed using mRS through face‐to‐face interviews or telephone follow‐ups by trained researchers, 3‐month functional independence was defined as a mRS score of ≤ 2 and a poor outcome was defined as a mRS score of > 3.

### 2.4 Clinical Data

Baseline demographic and medical history data were prospectively collected, including age, sex, history of hypertension, history of diabetes, prior acute ischemic stroke (AIS), prior ICH, alcohol use, smoking, the use of antiplatelet agents and anticoagulants within the one month before the ICH onset, and pre‐stroke mRS score. The admission systolic blood pressure (SBP), diastolic blood pressure (DBP), time to baseline imaging, and mode of transportation to the hospital were provided. Moreover, several severity scores, including the NIHSS score and GCS, were collected.

### 2.5 Imaging Analysis

All enrolled patients underwent head CT upon admission. Hematoma locations were classified into the thalamus, basal ganglia, or lobar regions, determined by the epicenter of the hematoma. Hematoma volumes were quantified using semiautomated computer‐assisted volumetric analysis (AnalyzeDirect, version 12.0) by trained researchers. Regions of interest were defined on axial CT slices, followed by threshold‐based segmentation to identify hemorrhagic areas and calculate total volume. Additionally, CT scans were assessed for IVH, subarachnoid hemorrhage (SAH), midline shift, and hydrocephalus.

### 2.6 Statistical Analysis

Continuous variables were reported as mean ± standard deviation (SD) for normally distributed data or as median (interquartile range, IQR) for non‐normally distributed data. Categorical variables were expressed as frequencies and percentages. For continuous variables, comparisons between two groups were performed using the Student's *t*‐test or Mann–Whitney *U* test based on data distribution, while one‐way ANOVA was used for comparisons among three groups. Group comparisons for categorical variables utilized the χ^2^ test or Fisher's exact test, as appropriate. The Youden index was generated to determine the optimal location‐specific hematoma volume cutoffs for predicting END in the training cohort; these thresholds were rounded to the nearest integer. Sensitivity analyzes were performed by incorporating age, NIHSS score, and IVH presence as covariates in the models evaluating outcomes. Multivariable logistic regression was performed to predict END, including variables with *p* < 0.1 in univariable analyzes. Backward selection was used to identify the most parsimonious combination of predictors. Multicollinearity was assessed using the variance inflation factor (VIF), and variables with VIF > 5.0 were removed from the final model. Considering the correlation between GCS and NIHSS scores, only the NIHSS score was included in the logistic regression [16]. Similarly, the optimal NIHSS score cutoff was determined using the Youden index from the ROC curve and converted into a binary variable. Subsequently, we developed a novel location‐specific hematoma volume for early neurological deterioration (LIVED) score by rounding each variable's β coefficient from the multivariable logistic regression model in the training cohort. The effectiveness of the score in predicting END and 3‐month outcomes was measured using the area under the curve (AUC) with the DeLong test across the training, internal, and external validation cohorts. We further compared the performance of our score with previously proposed prediction tools for ICH. Statistical analyzes were conducted using SPSS (Version 25.0; IBM Corp, Armonk, NY, USA) and MedCalc (Version 20.022; MedCalc Software, Ostend, Belgium).

## 3 Results

**FIGURE 1:**
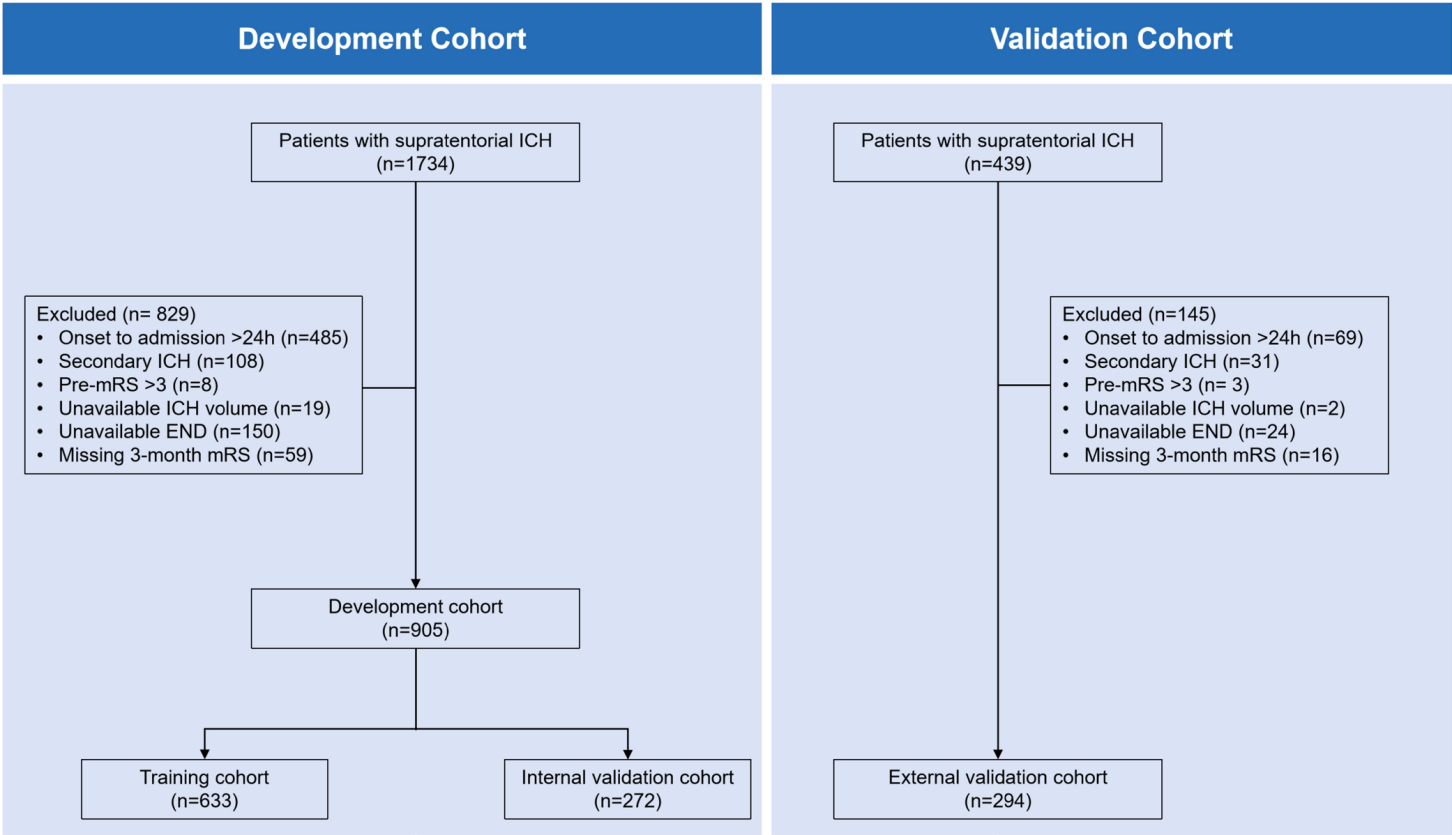
The flowchart of patient selection. END, early neurological deterioration; ICH, intracerebral hemorrhage; mRS, modified rankin scale.

A total of 1199 supratentorial ICH participants at two centers were enrolled, 905 patients from CQMU1H were further randomly split into training set (633 patients) and internal validation set (272 patients), and 294 patients from AHMU2H as external validation cohort. A detailed inclusion and exclusion flowchart was shown in Figure 1. The median age was 62 years (IQR, 52–71 years), and 68.6% were male. END occurred in 176 (14.7%) ICH patients within 24 h of admission. ICH location was basal ganglia in 662 (55.2%), thalamus in 281 (23.4%), and lobar in 256 (21.4%) of all participants. Characteristics and outcomes were comparable across all cohorts and were detailed in Table S1.

The comparison between patients with and without early neurological deterioration in the training cohort is presented in Table 1. Patients with END were more likely to have a history of AIS and presented with greater neurological severity, as indicated by lower GCS scores, higher NIHSS scores, and larger baseline hematoma volumes on admission. Additionally, they had a significantly higher incidence of IVH and midline shift. Furthermore, patients with END exhibited a lower rate of functional independence (17.4% vs. 56.6%, *p* < 0.001), a higher incidence of poor functional outcomes (72.8% vs. 29.6%, *p* < 0.001), and increased mortality rates (37.0% vs. 12.4%, *p* < 0.001) at 3 months.

**TABLE 1:** Comparison of characteristics between ICH patients with and without END in the training cohort.

Variables	Patients with END		*p*
	Yes (*n* = 92)	No (*n* = 541)	
Demographics			
Age (IQR), years	62 (50, 70)	62 (53, 70)	0.609
Male sex, *n* (%)	54 (58.7)	365 (67.5)	0.121
Transferred patients, *n* (%)	22 (23.9)	123 (22.7)	0.893
Medical history (at enrollment)			
Hypertension, *n* (%)	70 (76.1)	393 (72.6)	0.610
Diabetes, *n* (%)	20 (21.7)	94 (17.4)	0.380
Prior AIS, *n* (%)	14 (15.2)	39 (7.2)	0.015*
Prior ICH, *n* (%)	5 (5.4)	45 (8.3)	0.410
Previous antiplatelet agents, *n* (%)	7 (7.6)	27 (5.0)	0.312
Previous anticogulants, *n* (%)	0 (0.0)	6 (1.1)	0.601
Smoking, *n* (%)	32 (34.8)	213 (39.4)	0.419
Being drinking, *n* (%)	24 (26.1)	160 (29.6)	0.537
Clinical features			
Admission SBP (IQR), mmHg	172 (153, 192)	170 (152, 189)	0.389
Admission DBP (IQR), mmHg	99 (86, 110)	98 (86, 109)	0.945
Time from onset to first CT (IQR), h	3.6 (1.6, 11.1)	4.1 (1.8, 11.8)	0.251
NIHSS score (IQR)	14 (10, 19)	10 (4, 17)	< 0.001***
GCS score (IQR)	11 (9, 13)	14 (10, 15)	< 0.001***
CT imaging data			
ICH location, *n* (%)			0.589
Basal ganglia	52 (56.5)	315 (58.2)	
Thalamus	16 (17.4)	109 (20.1)	
Lobar	24 (26.1)	117 (21.6)	
Right‐side lesion, *n* (%)	59 (64.1)	283 (52.3)	0.072
ICH volume, median (IQR), mL	25.9 (14.1, 47.8)	11.3 (5.7, 25.3)	< 0.001***
Midline shift, *n* (%)	48 (52.2)	137 (25.3)	< 0.001***
Hydrocephalus, *n* (%)	20 (21.7)	75 (13.9)	0.058
IVH presence, *n* (%)	46 (50.0)	176 (32.5)	0.001**
SAH presence, *n* (%)	17 (18.5)	73 (13.5)	0.256
Outcomes			
3‐month functional independence, *n* (%)	16 (17.4)	306 (56.6)	< 0.001***
3‐month poor outcome, *n* (%)	67 (72.8)	160 (29.6)	< 0.001***
3‐month mortality, *n* (%)	34 (37.0)	67 (12.4)	< 0.001***

*Note:* *p < 0.05, **p < 0.005, ***p < 0.001.

Abbreviations: AIS, acute ischemic stroke; CT, computed tomography; DBP, diastolic blood pressure; END, early neurological deterioration; GCS, Glasgow coma scale; ICH, intracerebral hemorrhage; IQR, interquartile range; IVH, intraventricular hemorrhage; NIHSS, national institutes of health stroke scale; SAH, subarachnoid hemorrhage; SBP, systolic blood pressure.

### 3.1 Location‐Specific Hematoma Volume Cutoffs for END

**FIGURE 2:**
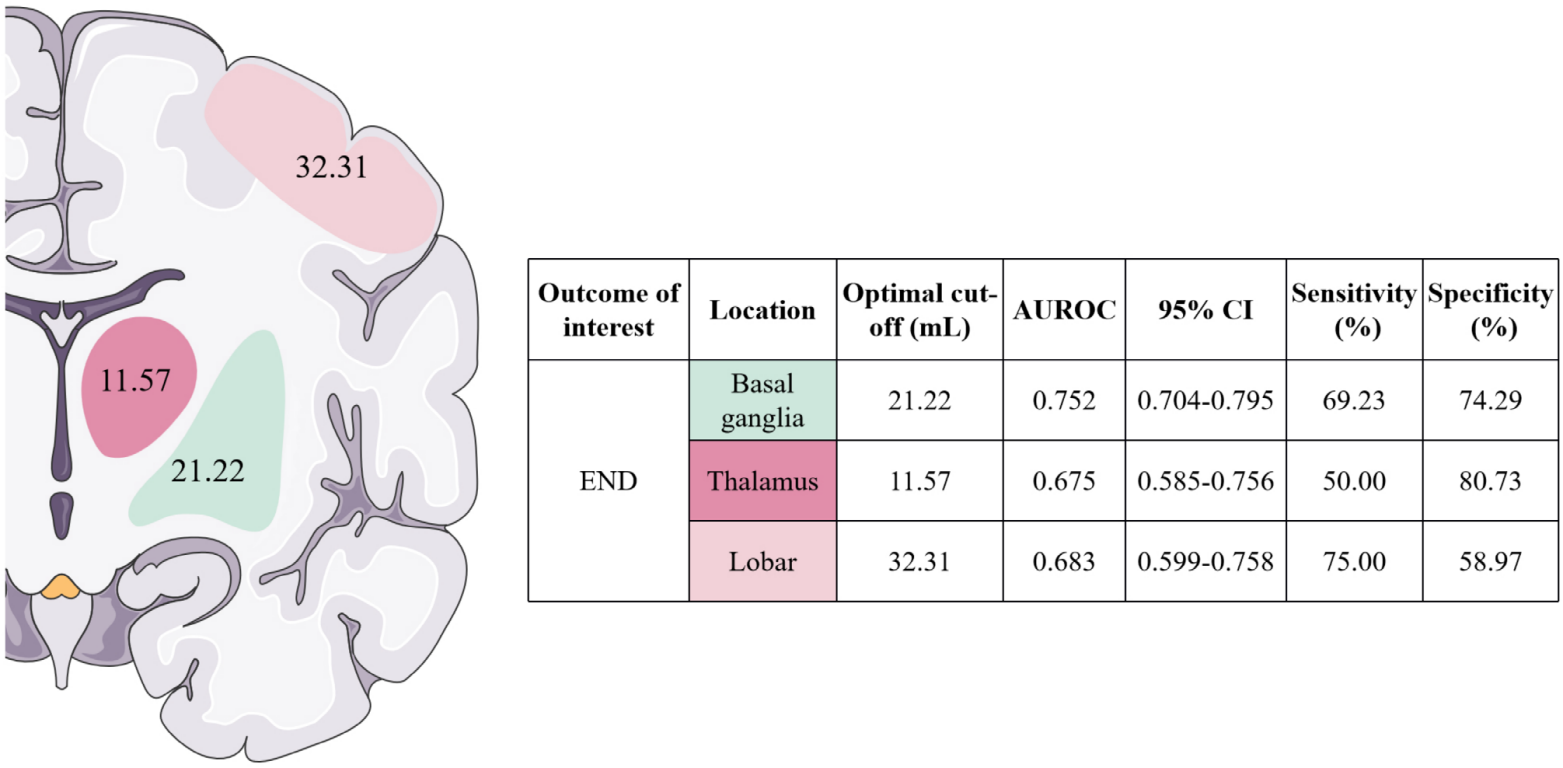
Receiver operating characteristics of hematoma volume for END of different locations in ICH. AUROC, area under the receiver operating characteristic; CI, confidence interval; END, early neurological deterioration; ICH, intracerebral hemorrhage.

The AUROC analysis evaluated the relationship between admission hematoma volume and the occurrence of END, stratified by location. Figure 2 presents the location‐specific hematoma volume cutoffs, along with corresponding sensitivity and specificity values. Optimal cutoff values for predicting END were determined to be 21 mL for basal ganglia, 12 mL for thalamus, and 32 mL for lobar hemorrhages. Multivariable logistic regression analysis revealed strong associations between the location‐specific hematoma volume cutoff and 3‐month outcomes, including functional independence (OR, 0.271; 95% CI, 0.164–0.446, *p* < 0.001), poor outcome (OR, 3.465; 95% CI, 2.133–5.629, *p* < 0.001), and mortality (OR, 3.162; 95% CI, 1.796–5.565, *p* < 0.001) (Table S2).

### 3.2 LIVED Score: A Simple Predictive Score for END

In the multivariable logistic regression analysis for END, after backward selection, the following factors remained in the model: location‐specific hematoma volume cutoff (OR, 4.15; 95% CI, 2.45–7.02, *p* < 0.001), NIHSS score (OR, 1.99; 95% CI, 1.10–3.59, *p* = 0.023), right‐side ICH (OR, 1.75; 95% CI, 1.07–2.87, *p* = 0.026), and prior AIS (OR, 2.31; 95% CI, 1.14–4.69, *p* = 0.020) (Table 2). All variables retained in the final model were incorporated into a predictive scoring model (Table 3).

**TABLE 2:** Multivariate analyzes of END in ICH patients.

Variable	Odds ratio	95% confidence interval	*p*
Location‐specific hematoma volume cutoff	4.15	2.45–7.02	< 0.001***
NIHSS score	1.99	1.10–3.59	0.023*
Right‐side ICH	1.75	1.07–2.87	0.026*
Prior AIS	2.31	1.14–4.69	0.020*

*Note:* *p < 0.05, **p < 0.005, ***p < 0.001.

Abbreviations: AIS, acute ischemic stroke; END, early neurological deterioration; ICH, intracerebral hemorrhage; NIHSS, national institutes of health stroke scale.

**TABLE 3:** Determinants of the LIVED score.

Parameters	Value		Points
Location‐specific hematoma volume cutoff	Basal ganglia	Hematoma volume ≤ 21 mL	0
		Hematoma volume > 21 mL	2
	Thalamus	Hematoma volume ≤ 12 mL	0
		Hematoma volume > 12 mL	2
	Lobar	Hematoma volume ≤ 32 mL	0
		Hematoma volume > 32 mL	2
NIHSS score	< 9		0
	≥ 9		1
Prior AIS	No		0
	Yes		1
Right‐side ICH	No		0
	Yes		1
Total score			5

Abbreviations: AIS, acute ischemic stroke; END, early neurological deterioration; ICH, intracerebral hemorrhage; NIHSS, national institutes of health stroke scale.

**FIGURE 3:**
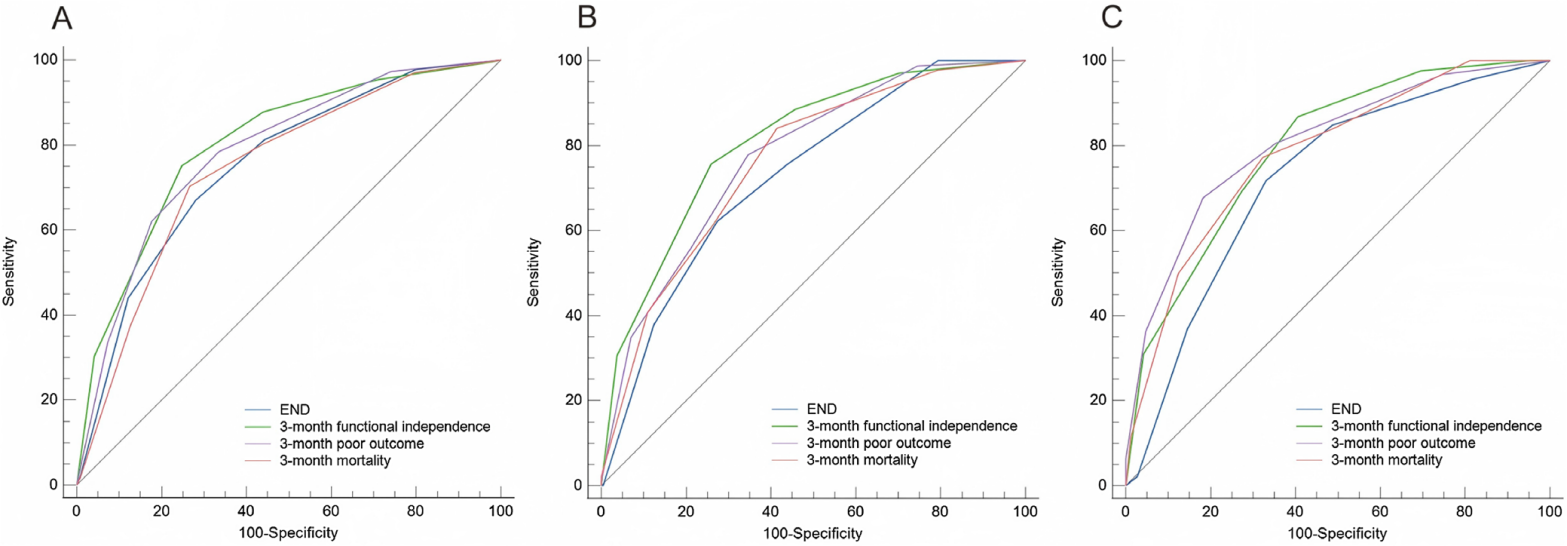
Receiver operating characteristic curve analysis of LIVED score for predicting END and 3‐month outcomes in (A) training cohort, (B) internal validation cohort, and (C) external validation cohort. END, early neurological deterioration; LIVED, location‐specific hematoma volume for early neurological deterioration.

The LIVED score demonstrated strong predictive performance for END with AUC values of 0.755, 0.729 and 0.723 in the training, internal validation, and external validation cohorts, respectively. The LIVED score also showed robust predictive ability for 3‐month outcomes, including functional independence, poor outcome, and mortality, consistently across training, internal validation, and external validation cohorts (Table S3). Figure 3 shows a visualization of ROC curves for the LIVED score in predicting clinical outcomes.

**FIGURE 4:**
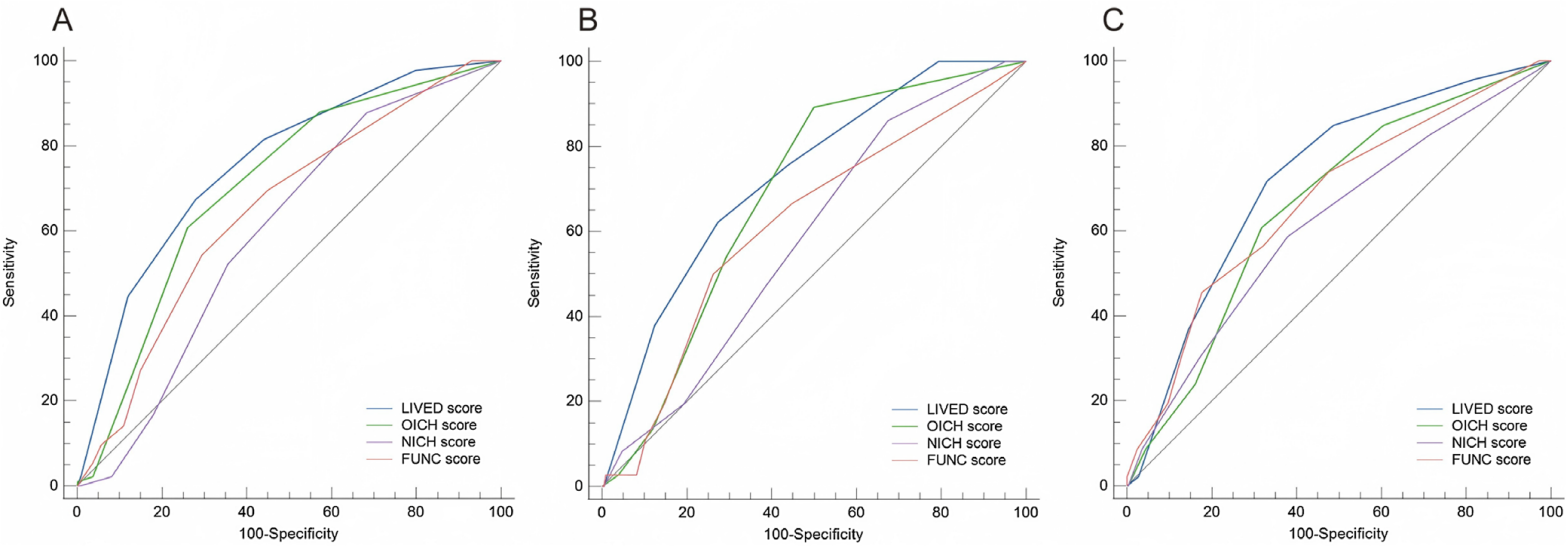
Receiver operating characteristic curve analysis of LIVED score and other ICH scores for predicting END in (A) training cohort, (B) internal validation cohort, and (C) external validation cohort. END, early neurological deterioration; ICH, intracerebral hemorrhage; LIVED, location‐specific hematoma volume for early neurological deterioration; NICH, new intracerebral hemorrhage; OICH, original intracerebral hemorrhage.

We compared the performance of the LIVED score with three previously established ICH scores: the original ICH (OICH) score, the new ICH (NICH) score, and the FUNC score [17, 18, 19]. The LIVED score demonstrated superior discriminatory ability for END, achieving the highest AUC in all three cohorts. In the training cohort, the LIVED score achieved an AUC of 0.755, compared to 0.699 (OICH score), 0.556 (NICH score), and 0.651 (FUNC score). Figure 4 illustrates the ROC curves for all compared scores in the three cohorts, with the detailed AUC values provided in Table S4.

## 4 Discussion

In this large, two‐stage study of patients with supratentorial ICH, we established location‐specific hematoma volume thresholds that independently predict early neurological deterioration. Building on these findings, we proposed the LIVED score, which integrates four readily available clinical and radiological variables into a simple 0–5 scale. The LIVED score demonstrated consistent and clinically meaningful discrimination and calibration for both END and 3‐month outcomes, with stable performance across the derivation, internal, and external validation cohorts.

Our study contributes to the establishment of optimal cutoff values for location‐specific hematoma volumes, offering a new tool to independently predict END in patients with supratentorial ICH. These cutoff values were significantly associated with END and further validated for their correlation with clinical outcomes. Specifically, the identified cutoff values independently predicted functional independence (mRS 0–2), poor prognosis (mRS 4–6), and mortality at 3 months. Although hematoma volume is a well‐established predictor of outcomes, increasing evidence indicates that its prognostic impact varies by location. Ironside N and colleagues reported that outcomes after ICH vary according to location‐specific hematoma volume thresholds, and that incorporating these thresholds into a modified ICH score yielded excellent discrimination for 90‐day functional dependence and mortality [11]. Similarly, another study identified optimal hematoma volume cutoffs for different ICH locations in predicting 6‐month outcomes, including good outcomes, poor outcomes and mortality, emphasizing the influence of location‐specific hematoma size on prognosis [10]. A recent study further explored the interaction between final ICH volume and outcomes by location, noting a lower volume tolerance in deep ICH and reporting that hematoma expansion significantly impacts poor functional outcomes only when the final ICH volume exceeds a critical threshold [12]. In alignment with these findings, our established cutoff values of location‐specific hematoma volumes provide a robust and validated tool for predicting END and 3‐month outcomes in supratentorial ICH, enhancing prognostic precision in clinical practice.

The initial NIHSS score is widely recognized as a robust predictor of both functional outcomes and END. Consistent with prior studies, our results confirm that higher NIHSS scores are associated with an increased likelihood of END, reflecting the greater severity of stroke in these patients. Previous research has identified prior ICH as a risk factor for END; our study further established a history of AIS as an independent predictor of END [20]. A possible explanation is that prior stroke may heighten the brain's vulnerability to subsequent stroke events, thereby exacerbating neurological decline. Existing studies have reported that right hemispheric ICH is associated with a higher risk of mortality directly attributable to the initial hemorrhage [21]. Consistent with this observation, we found that patients with right‐sided ICH were more likely to experience END. An intriguing explanation for this association may relate, at least in part, to the definition of END, which is based on short‐term changes in the NIHSS and GCS scores. Both scales incorporate items that are disproportionately influenced by language‐related functions. In left‐hemispheric ICH, early language impairment is more common and may reduce the sensitivity of these scales to detect subtle neurological worsening over a short time interval. Consequently, deterioration occurring in non‐language domains may be partially masked. In contrast, in right‐sided ICH, where language function is less frequently involved, END may be more readily captured by variations in NIHSS and GCS scores. This scale‐related asymmetry in detecting neurological deterioration may therefore partially contribute to the observed independent association between right‐sided ICH and END. Given that END occurs within 24 h of hospital admission, our study focused exclusively on baseline variables that were readily available at presentation. Variables such as hematoma expansion, which typically manifests within 24 h and poses challenges in establishing a clear temporal relationship with END, were not incorporated into the analyze [22]. This approach ensured that our predictive model relied on immediately accessible clinical data, enhancing its practical applicability in the acute management of ICH.

Most existing predictive models for ICH have traditionally focused on long‐term outcomes [17, 18, 19]. The ICH score integrates the GCS, ICH volume, IVH, infratentorial origin, and age to estimate 30‐day mortality. Building upon this, the NICH score incorporates the NIHSS, IVH, subarachnoid extension, admission temperature, and pulse pressure to predict both mortality and favorable functional outcomes. Similarly, the FUNC score, tailored for functional prognosis, combines pre‐ICH cognitive impairment with acute clinical variables to forecast 90‐day outcomes. Given the severe and rapidly evolving nature of ICH, the early identification of patients at high risk for END is critical for guiding timely interventions and optimizing clinical outcomes. Recent studies, including the Code ICH framework, emphasized the importance of early management and intervention in ICH patients to improve outcomes [23]. Furthermore, a growing body of evidence supports the benefits of early therapeutic interventions, such as intensive blood pressure management and anticoagulation reversal, in mitigating ICH progression [24, 25, 26]. To address this need, we propose a novel scoring system, the LIVED score, specifically designed to predict END following ICH. In addition to its primary role in predicting END, the LIVED score also effectively predicts 3‐month outcomes—including functional independence, poor outcome, and mortality—thus providing a comprehensive framework for both immediate and extended ICH prognostication. In comparison with the previous OICH score, NICH score, and FUNC score, the LIVED score showed improved discrimination for the prediction of END of ICH [17, 18, 19]. The reliability and generalizability of the LIVED score have been validated across the training, internal validation, and external validation cohorts, where it consistently demonstrated robust predictive performance.

The LIVED score demonstrates moderate predictive performance for END, which may limit its use for individual‐level prognostication. Nevertheless, it holds promise for aiding clinical decision‐making in acute care settings when used as part of a broader clinical assessment. In acute care settings, rapid identification of patients at high risk for END is essential for guiding early interventions such as intensive blood pressure control, reversal of anticoagulation, or early neurocritical care consultation [17, 18, 19, 22]. The LIVED score may assist front line providers by offering a structured approach to risk stratification at admission, potentially informing decisions regarding closer monitoring or escalation of care, depending on the clinical context and resource availability. Conversely, lower‐risk patients might be considered for step‐down care or participation in trials targeting neurological stability. While its use should not replace clinical judgment, the score could support triage decisions, especially in resource‐limited settings where ICU beds or neurosurgical capabilities are scarce. Implementation of the LIVED score into electronic health records or mobile applications could enable real‐time calculation and integration into standardized stroke care pathways, enhancing consistency and efficiency of care. In multicenter networks or stroke telemedicine platforms, LIVED scoring could help prioritize inter‐hospital transfers and optimize use of tertiary‐level stroke resources. From a research standpoint, the LIVED score could also serve as an enrichment tool for identifying high‐risk populations in future clinical trials aimed at reducing END. By stratifying patients based on early risk, clinical studies may achieve better statistical power and target those most likely to benefit from intervention.

While the LIVED Score has demonstrated stable predictive performance, several limitations warrant consideration. As this was a retrospective study, inherent selection and information biases cannot be excluded. Furthermore, our study was limited to patients with supratentorial ICH, and the applicability of our findings to patients with infratentorial hemorrhage remains to be determined. Although we validated the score in an external cohort, its moderate predictive performance highlights the need for further prospective, multicenter studies to assess its generalizability across diverse populations, care settings, and imaging protocols.

In conclusion, we developed and independently validated a location‐specific hematoma volume assessment for predicting END and introduced the novel LIVED score, a practical tool demonstrating robust generalizability and reliable predictive performance for END and 3‐month outcomes in supratentorial ICH.

## Author Contributions

Zuoqiao Li and Qi Li conceptualized and designed the study. Zuoqiao Li and Guilin Meng conducted data analysis and interpretation. Zuoqiao Li drafted the manuscript. Zuoqiao Li, Zijie Wang, Xiao Hu, Jing Cheng, Chu Chen, Tiannan Yang, Jiaxin Luo, Zizhen Meng, and Xueyun Liu performed data collection. Qi Li supervised the study. Qi Li and Xueyun Liu secured funding. All authors reviewed and approved the final manuscript.

## Funding

This work was supported by National Natural Science Foundation of China (Grant 82471368), Excellent Research and Innovation Team Project of Anhui Province (Grant 2024AH010014), Research Fund of Anhui Institute of Translational Medicine (Grant 2022zhyx‐C38), Clinical and Translational Research Project of Anhui Province (Grants 202427b10020053 and 202427b10020090), and Health Research Program of Anhui (Grant 2024Aa40015).

## Ethics Statement

The study was conducted in accordance with the recommendations of the Declaration of Helsinki and was approved by the Ethics Committee of the First Affiliated Hospital of Chongqing Medical University (2025‐413‐01) and the Ethics Committee of the Second Affiliated Hospital of Anhui Medical University (YX2025‐174).

## Consent

Written informed consent was waived due to the retrospective design of the study.

## Conflicts of Interest

The authors declare no conflicts of interest.

## Supporting information


**Table S1:** Comparison of characteristics among training, internal validation, and external validation study cohort.
**TABLE S2:** Association between location‐specific hematoma volume cutoff and outcomes of interest.
**TABLE S3:** AUC of LIVED score in predicting different outcomes after supratentorial ICH.
**TABLE S4:** AUC of LIVED score and other ICH scores in predicting END after supratentorial ICH.

## Data Availability

The data that support the findings of this study are available on request from the corresponding author. The data are not publicly available due to privacy or ethical restrictions.
