# Supplementary material for: Location‐Specific Hematoma Volume Predicts Early Neurological Deterioration in Supratentorial ICH
Source: Ann Clin Transl Neurol. 2026 Feb 22;13(8):1653–61. doi: 10.1002/acn3.70351 (PMC13394142; doi:10.1002/acn3.70351)
Supplement: Supplementary file 1 [file ACN3-13-1653-s001.docx]

Location-Specific Hematoma Volume Predicts Early Neurological Deterioration in Supratentorial ICH

**Supplemental Material**

Table S1. Comparison of characteristics among training, internal validation, and external validation study cohort.

| **Variables** | **Training cohort**  **(n = 633)** | **Internal validation cohort (n = 272)** | **External validation cohort (n = 294)** | ***p* Value** |
| --- | --- | --- | --- | --- |
| **Demographics** | | | | |
| Age (IQR), years | 62 (52, 70) | 61 (51, 71) | 60 (52, 71) | 0.724 |
| Male sex, n (%) | 419 (66.2) | 197 (72.4) | 206 (70.1) | 0.149 |
| Transferred patients, n (%) | 145 (22.9) | 63 (23.2) | 65 (22.1) | 0.961 |
| **Medical history (at enrollment)** | | | |  |
| Hypertension, n (%) | 463 (73.1) | 204 (75.0) | 243 (82.7) | 0.010* |
| Diabetes, n (%) | 114 (18.0) | 48 (17.6) | 50 (17.0) | 0.904 |
| Prior AIS, n (%) | 53 (8.4) | 19 (7.0) | 54 (18.4) | < 0.001*** |
| Prior ICH, n (%) | 50 (7.9) | 20 (7.4) | 20 (6.8) | 0.829 |
| Previous antiplatelet agents, n (%) | 34 (5.4) | 14 (5.1) | 37 (12.6) | < 0.001*** |
| Previous anticogulants, n (%) | 6 (0.9) | 3 (1.1) | 6 (2.9) | 0.417 |
| Smoking, n (%) | 245 (38.7) | 131 (48.2) | 73 (24.8) | < 0.001*** |
| Drinking, n (%) | 184 (29.1) | 87 (32.0) | 62 (21.1) | < 0.001*** |
| **Clinical features** | | | |  |
| Admission SBP (SD), mmHg | 170 (152,190) | 170.0 (154,187) | 173 (152,195) | 0.205 |
| Admission DBP (SD), mmHg | 98 (86,110) | 99 (87,111) | 101 (91,114) | 0.022* |
| Time from onset to first CT (IQR), h | 4.0 (1.8, 11.7) | 4.4 (1.9, 11.9) | 4.1 (1.9, 10.7) | 0.056 |
| NIHSS score (IQR) | 10 (5,18) | 10 (5,17) | 10 (4,17) | 0.906 |
| GCS score (IQR) | 14 (10,15) | 14 (11,15) | 15 (10,15) | 0.681 |
| END, n (%) | 92 (14.5) | 38 (14.0) | 46 (15.6) | 0.852 |
| **CT imaging data** | | | | |
| ICH location, n (%) |  | | | 0.007** |
| Basal ganglia | 367 (58.0) | 144 (52.9) | 151 (51.4) |  |
| Thalamus | 125 (19.7) | 66 (24.3) | 90 (30.6) |  |
| Lobar | 141 (22.3) | 62 (22.8) | 53 (18.0) |  |
| Right-side lesion, n (%) | 342 (54.0) | 140 (51.5) | 139 (47.3) | 0.225 |
| ICH volume, median (IQR), mL | 12.6 (6.2,28.1) | 12.3 (5.9,24.8) | 14.5 (6.3,34.2) | 0002** |
| Midline shift, n (%) | 185 (29.2) | 69 (25.4) | 59 (20.1) | 0.012* |
| Hydrocephalus, n (%) | 95 (15.0) | 36 (13.2) | 38 (12.9) | 0.632 |
| IVH presence, n (%) | 222 (35.1) | 98 (36.0) | 119 (40.5) | 0.275 |
| SAH presence, n (%) | 90 (14.2) | 32 (11.8) | 30 (10.2) | 0.213 |
| **Outcomes** | | | | |
| 3-month functional independence, n (%) | 322 (50.9) | 140 (51.5) | 129 (43.9) | 0.100 |
| 3-month poor outcome, n (%) | 227 (35.9) | 87 (32.0) | 124 (42.2) | 0.037* |
| 3-month mortality, n (%) | 101 (16.0) | 45 (16.5) | 44 (15.0) | 0.875 |

Abbreviation: AIS, Acute Ischemic Stroke; CT, Computed Tomography; DBP, Diastolic Blood Pressure; END, Early Neurological Deterioration; GCS, Glasgow Coma Scale; ICH, Intracerebral Hemorrhage; IQR, Interquartile Range; IVH, Intraventricular Hemorrhage; NIHSS, National Institutes of Health Stroke Scale; SAH, Subarachnoid Hemorrhage; SBP, Systolic Blood Pressure.

Table S2. Association between location-specific hematoma volume cutoff and outcomes of interest.

| **Variable** | **Outcomes of interest** | **ICH volume larger than the cutoff** | |
| --- | --- | --- | --- |
|  |  | **Adjusted OR (95%CI)** | ***p* Value** |
| Location-specific hematoma volume cutoff | 3-month functional independence* | 0.271 (0.164-0.446) | <0.001 |
|  | 3-month poor outcome* | 3.465 (2.133-5.629) | <0.001 |
|  | 3-month mortality* | 3.162 (1.796-5.565) | <0.001 |

*Adjustment for age, NIHSS score, IVH presence.

Abbreviation: CI, Confidence Interval; ICH, Intracerebral Hemorrhage; OR, Odds Ratio.

Table S3. AUC of LIVED score in predicting different outcomes after supratentorial ICH.

| **Outcomes of Interest** | **AUC** | **95% Confidence Interval** | **Sensitivity (%)** | **Specificity (%)** |
| --- | --- | --- | --- | --- |
| **Training cohort** | | | | |
| END | 0.755 | 0.720-0.788 | 67.39 | 72.09 |
| 3-month functional independence | 0.803 | 0.769-0.833 | 75.16 | 75.24 |
| 3-month poor outcome | 0.785 | 0.751-0.816 | 78.41 | 66.50 |
| 3-month mortality | 0.745 | 0.710-0.779 | 70.30 | 73.31 |
| **Internal validation cohort** | | | | |
| END | 0.729 | 0.672-0.781 | 62.16 | 72.65 |
| 3-month functional independence | 0.807 | 0.755-0.852 | 75.71 | 74.05 |
| 3-month poor outcome | 0.773 | 0.718-0.821 | 77.91 | 65.41 |
| 3-month mortality | 0.761 | 0.705-0.810 | 84.09 | 58.59 |
| **External validation cohort** | | | | |
| END | 0.723 | 0.668-0.773 | 71.74 | 66.94 |
| 3-month functional independence | 0.794 | 0.743-0.839 | 86.82 | 59.39 |
| 3-month poor outcome | 0.805 | 0.756-0.849 | 67.74 | 81.76 |
| 3-month mortality | 0.780 | 0.729-0.826 | 77.27 | 67.60 |

Abbreviation: AUC, Area Under the Curve; END, Early Neurological Deterioration; ICH, Intracerebral Hemorrhage; LIVED, Location-specIfic hematoma Volume for Early neurological Deterioration.

Table S4. AUC of LIVED score and other ICH scores in predicting END after supratentorial ICH.

| **Outcomes of Interest** | **AUC** | **95% Confidence Interval** | **Sensitivity (%)** | **Specificity (%)** |
| --- | --- | --- | --- | --- |
| **Training cohort** | | | | |
| LIVED score | 0.755 | 0.720-0.788 | 67.03 | 71.96 |
| OICH score | 0.699 | 0.662-0.735 | 59.78 | 73.75 |
| NICH score | 0.556 | 0.517-0.596 | 48.35 | 64.25 |
| FUNC score | 0.651 | 0.613-0.688 | 70.65 | 54.90 |
| **Internal validation cohort** | | | | |
| LIVED score | 0.729 | 0.672-0.781 | 62.16 | 72.65 |
| OICH score | 0.687 | 0.628-0.742 | 89.47 | 50.43 |
| NICH score | 0.597 | 0.536-0.656 | 51.35 | 64.35 |
| FUNC score | 0.612 | 0.551-0.670 | 50.00 | 73.08 |
| **External validation cohort** | | | | |
| LIVED score | 0.723 | 0.668-0.773 | 71.74 | 66.94 |
| OICH score | 0.648 | 0.589-0.704 | 62.22 | 64.96 |
| NICH score | 0.613 | 0.553-0.671 | 55.56 | 63.68 |
| FUNC score | 0.649 | 0.590-0.705 | 46.67 | 79.49 |

Abbreviation: AUC, Area Under the Curve; END, Early Neurological Deterioration; ICH, Intracerebral Hemorrhage; LIVED, Location-specIfic hematoma Volume for Early neurological Deterioration; NICH, New Intracerebral Hemorrhage; OICH, Original Intracerebral Hemorrhage.
